# Medical Miscellany

**Published:** 1853-10

**Authors:** 


Medical Miscellany.—It appears by the U. S. Census, that the consumption of spirituous and malt liquors reaches the enormous quantity of eighty-six millions of gallons annually, equal to six gallons for every adult person.——A woman in New York, recently died from convulsions, produced by eating shad.—Mons. Roulin produces colored silk, of different hues, by feeding dyeing materials to the worms, with the mulberry leaves.......A convention of some seventy or eighty deaf mutes was recently held at Montpelier, Vermont, most of whom were from that State.——The cultivation of the camphor plant has recently been introduced into Louïsiana.——The best mannikins are made in France: they are various sizes and prices. About 18 inches high for $90: 4 feet high, with 1700 pieces, at $350: same size, with 1200 parts, for $200; 6 feet high, with 1200 pieces, $400; but with 1700 parts, for $950.——The N. Y. Medical Gazette states that 17 suicides, 5 murders, and 209 cases of insanity are directly traceable to "spiritual" excitement, as the cause.——Owen Duffy, of Ireland, is 122 years old. When 116 he lost his second wife, and soon married a third, by whom he had a son and daughter. His oldest *boy* is 90 years old. His mental and corporeal faculties are still vigorous, and he walks a distance of eight miles.——Prince Albert has lately had the measles. ——Dr. Marshall Hall, who recently made a tour through the United States, has since traveled through Canada.——A man in New York City, known as Dr. Watts, was fined $1100, for injury inflicted on a child, by giving a compound of his manufacture, called Watts' Nervous Antidote.——The human voice has been heard across the Straits of Gibralter, a distance of ten miles: it only happens in peculiar states of weather. The sound of a military band has been heard 70 miles on a clear frosty morning.——Edward Cranson, the Kentish giant, said to be the largest boned man in Europe, measures 7 feet 6 inches, weighs 490 lbs., can reach perpendicularly 10 feet 6 inches, and is under 21 years of age.——Drs. Mott, of New York City, and Warren, of Boston, have been elected members of the Academie de Medecine, of Paris.——Mrs. Blanchard, of Ticonderoga, aged 78 years, has lately cut a new set of teeth, says the Dental News Letter.——The Ulster (N. Y.) Rep. says that Mr. Vroman, of western New York, went to sleep in 1848, and has slept most of the time till now. He has had, during his five years' sleep, a few waking periods, the longest, sixteen hours. He is a mere skeleton: breathes freely, perspires copiously, and sleeps always on his left side. His jaws are set; his teeth are pried open daily to feed him on milk, a quart of which a day, with a little bread, is his food. Whenever he does wake up, he always calls, at once, for "something to eat."

